# Interfascial Injection Pressure Depending on Type of Regional Anesthesia Needle

**DOI:** 10.3390/jcm15041458

**Published:** 2026-02-12

**Authors:** Wilk Mateusz, Jedrasiak Karol, Suwalska Aleksandra, Wodarski Piotr

**Affiliations:** 1 Collegium Medicum, WSB University, 41-300 Dabrowa Gornicza, Poland; 2 Department of Anesthesiology and Intensive Care, University Clinical Center, 40-055 Katowice, Poland; 3 Department of Transport and Computer Science, WSB University, 41-300 Dabrowa Gornicza, Poland; 4 Department of Data Science and Engineering, Faculty of Automatic Control, Electronics and Computer Science, Silesian University of Technology, 44-100 Gliwice, Poland; 5 Department of Biomechatronics, Faculty of Biomedical Engineering, Silesian University of Technology, 44-100 Gliwice, Poland

**Keywords:** interfascial space, needle, pressure, injection, regional anesthesia

## Abstract

**Introduction:** Reliable identification regarding interfascial spaces proves essential to achieve successful nerve block analgesia; however, ultrasound guided approaches are recognized as challenging, particularly in obese or pediatric patients. In prior cadaveric and clinical investigations, multiple approaches were evaluated to identify methods for measuring injection pressures as a function of needle position relative to fascia. Our previous study proposed simpler method of finding interfascial spaces with the needle tip. In this study, it was examined whether needle tip design influences injection pressures during regional anesthesia procedures, via an ex vivo pig specimen setup. **Methods:** A bespoke apparatus for tracking injection pressure was deployed to enable continuous measurement of intraluminal pressure generated while delivering saline throughout ultrasound guided peripheral nerve block needles conducted within pig thigh specimens. Delivery was performed using an infusion pump. Three types of needles of the same manufacturer (Pajunk) and same diameter (22G) but with different tips (Facet, Facet S and Sprotte) were used to measure injection pressures during penetration through tissues until interfascial plane hydrodissection was created. Statistical analyses were performed to compare pressure levels, variability, and temporal pressure trends. **Results:** Ninety ultrasound guided injections in porcine thigh tissue were analyzed, with thirty procedures per needle type. Injection pressure differed significantly between intramuscular, fascial puncture, and interfascial phases, showing a distinct puncture peak (*p* ≤ 3.44 × 10^−14^). Needle geometry significantly affected pressures across all phases (Kruskal–Wallis intramuscular *p* = 2.0 × 10^−6^, puncture *p* = 7.52 × 10^−8^, interfascial *p* = 9.2 × 10^−5^), with large pairwise effects (Hedges g up to 1.51). The classical tip produced the highest intramuscular and higher interfascial pressures, the sharp tip required the lowest puncture pressure, and the lateral tip yielded the lowest intramuscular and interfascial pressures. **Conclusions:** Needle tip geometry substantially influences pressure dynamics throughout the injection process, with the classical design associated with the highest fascia-penetration injection pressures and the sharp needle exhibiting the lowest, while the lateral design associated with lowest intramuscular-penetration and interfascial pressures.

## 1. Introduction

Regional blocks are commonly categorized into plexus and peripheral nerve blocks as well as interfascial plane blocks. In interfascial plane blocks, local anesthetic is injected into the space between two layers of muscle fasciae without direct targeting of specific nerves or plexuses, with the intent of covering extended interfascial planes where nerves are located [1]. In selected patient groups, fascia is frequently characterized as tough and difficult to penetrate with a needle, including obese patients, individuals affected by long lasting immune mediated fibrous tissue disorders and individuals experiencing persistent nociception [2,3]. In such circumstances, inadvertent passage through the fascial compartment into muscle may occur, which can lead to ineffective blockade and local anesthetic induced myotoxicity [4]. In pediatric patients, difficulties in precise localization of the needle tip within the interfascial space may be encountered because pediatric fasciae are generally more elastic than adult fasciae [5]. In obese patients, ultrasound supported regional blocks are considered particularly demanding because increased tissue thickness and ultrasound attenuation reduce interfascial plane visibility, thereby limiting needle tip guidance [6]. In previous studies, opening injection pressures above 15 PSI, where PSI denotes pound per square inch, corresponding to 103.42 kPa, were reported to indicate intraneural injection of local anesthetic [7,8,9,10]. Those studies, however, did not take into consideration the pressure built inside tubing during injection and as a result did not show pressures produced by the tissues themselves, but rather by tissues and measurement system. In 2016, a study by Gadsden J.C. [11] showed that needle tip contact with a fascia produced opening injection pressure >15 PSI (=103.42 kPa), followed by Quadri C. [12] who proposed the first continuous injection pressure measurement system, based on a pressure fiber optic sensor placed inside the needle shaft just after needle tip. This system was later evaluated and used in further studies, which showed significant differences between pressure at the very needle tip and inside the needle shaft and tubing, focusing on the need of needle tip pressure monitoring [13,14,15,16]. Then, we performed a study [17] which proposed much simpler, but reliable method of both finding interfascial space and measuring pressures throughout needle penetration (in muscle, indenting fascia and during opening of interfascial space), which was also based on measurement of needle tip pressure but in relation to plateau pressure measured before puncture of the tissues. In this study, we investigated whether a type of needle tip influences injection pressures during regional anesthesia procedures.

The study aims to better understand the pressures associated with the continuous injection of saline to tissues through needles with different tip shapes but the same diameter. If such differences were to be demonstrated, it would indicate that the approach used to measure the injection pressure of local anesthetics needs to be modified.

## 2. Materials and Methods

Prior to initiating experiments, authorization was obtained from the Local Ethical Committee for Animal Experiments of Medical University of Silesia in Katowice, Poland, was obtained (BNW/NWN/0052/LKE/4/24).

**Figure 1:**
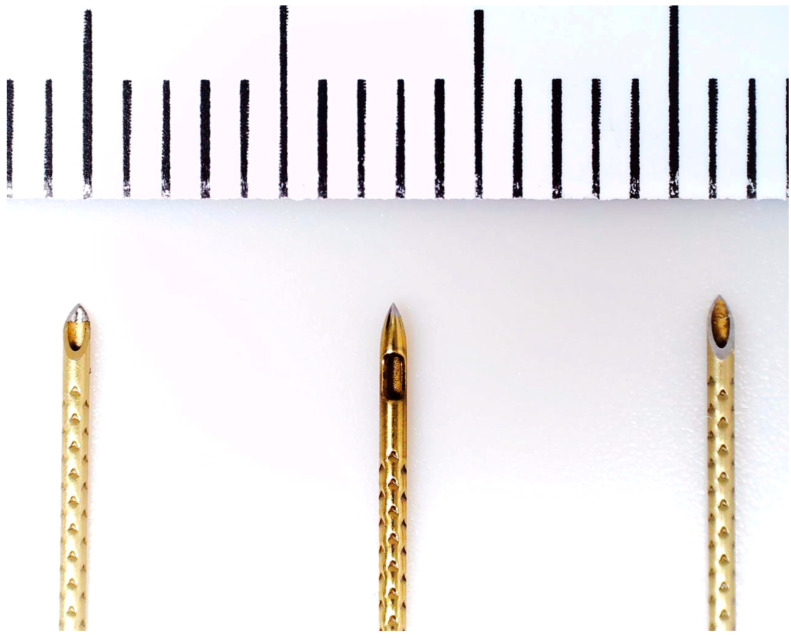
Images of needle tips. (**Left**): Pajunk SonoPlex II (classical); (**Center**): Pajunk SonoPlex II Sprotte (lateral) (**Right**): Pajunk Sonoplex II Facet S (sharp).

Pressure measurements were obtained using three types of regional anesthesia needles that differed in geometry and fluid delivery characteristics: Pajunk SonoPlex II 22G × 80 mm (classical in text) (Pajunk Medical Systems, Alpharetta, GA, USA), Pajunk SonoPlex II Facet S 22G × 80 mm (sharp in text) and SonoPlex II Sprotte 22G × 90 mm (lateral in text) (Figure 1). The measurements were performed using the system described earlier in [17], but instrumentation comprised commercial volumetric driver, single use syringe 60 mL, two 150 cm lines, triport valve, sensing module, and perineural cannula. Derived readouts were forwarded toward host workstation through universal serial bus link, using the chosen sampling cadence. Switch state signals accompanied readouts, entering the control software.

All porcine thighs were harvested from a slaughterhouse on the morning of the test day. No more than 10 h passed between obtaining the porcine thighs and performing tests. All tests were conducted at room temperature.

Throughout experimentation, 0.9% sodium chloride delivery proceeded without interruption via volumetric driver set at 10 mL/min (600 mL/h), preserving earlier protocol [17]. Signal capture for volumetric administration plus line load readouts commenced prior to bolus delivery, persisted amid cannula repositioning inside specimen material plus liquid assisted cleavage between fascia sheets, then ceased upon cannula withdrawal, leaving specimen material. Opening phase evaluated baseline (plateau) pressure was produced via apparatus with cannula held outside specimen material. Once recorded trace reached steady state, cannula advanced within specimen material, oriented at fascia interface. Across protocol duration, 0.9% sodium chloride solution flowed constantly via volumetric driver, permitting line load tracking while cannula progressed across successive anatomic strata.

Following identification of fascia interface by cannula bevel—or by lateral aperture near bevel—a 60 s interval was counted to quantify load within that compartment. Across protocol, 10 mL 0.9% sodium chloride solution entered each compartment. One separated interfascial hydrodissection was performed in different, randomly selected interfascial planes in every thigh. For each needle, 30 independent experimental trials were conducted under identical physical and procedural conditions to ensure comparability of results. During the study, 90 porcine thighs were used. An anaesthesiologist executed ultrasound guided in plane entry through swine thigh tissues, steering needle tip toward randomly chosen interfascial space until liquid-assisted separation between neighboring muscle fasciae became evident (kayak sign). The operator only saw the image on the ultrasound machine and the results of the measurements were blinded; the moments of passing through the muscle, resting on the fascia, puncturing the fascia, and the hydrodissection of the interfascial space, as well as the exit of the needle from the fascial space, were noted in the instrumentation control module. In general, methodology of needle penetration and continuous injection pressure monitoring were the same as in our previous study, with some exceptions such as a new portable ultrasound probe—Mindray TEAir—was used and three types of ultrasound needles—Pajunk SonoPlex II 22G × 80 mm, Pajunk SonoPlex II Facet S 22G × 80 mm and SonoPlex II Sprotte 22G × 90 mm—were used. This study is a continuation of our previous, pilot study.

**Figure 2:**
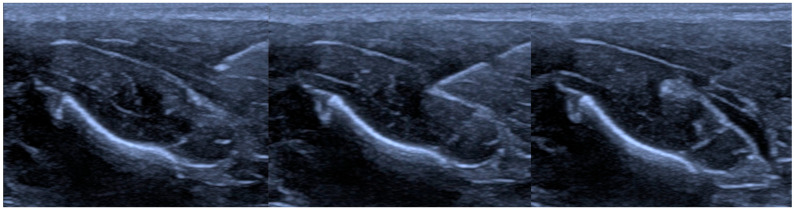
Stages of needle penetration through tissue. (**Left**): needle in muscle, (**Center**): fascia indentation, (**Right**): interfascial hydrodissection.

Ultrasound guidance and representative needle positioning are illustrated in Figure 2. The recorded pressure time series were quality controlled prior to analysis to ensure strictly increasing acquisition timestamps and to mitigate sampling artifacts attributable to intermittent device buffering. Where duplicated, missing, or out of order timestamps were detected, the affected samples were corrected using time-based alignment rules or they were excluded, so that subsequent computations reflected physiologic and mechanical changes rather than acquisition irregularities.

For each needle type, we first characterized the within trial pressure dynamics using complementary summary descriptors: mean normalized pressure, pressure variability quantified as the standard deviation, and the rate of pressure change quantified by the slope of a linear regression fitted to the relevant injection segments. To test whether these descriptors differed across repeated phases of the procedure within the same needle type, we selected the inferential framework based on distributional diagnostics. When parametric assumptions were satisfied, we applied repeated measures analysis of variance, followed by paired *t*-tests for planned phase comparisons, with multiplicity controlled using the Benjamini–Hochberg false discovery rate procedure. When normality was not supported, we used the Friedman test with Wilcoxon post hoc paired comparisons, again applying false discovery rate control. Phase specific differences in variability and slope were evaluated using paired *t*-tests or Wilcoxon signed rank tests according to normality. Effect magnitude was reported using Hedges g. In addition, we assessed systematic temporal tendencies within segments by testing whether the fitted slope differed from zero using one sample *t* tests or Wilcoxon signed rank tests, which provided a direct measure of consistency in pressure evolution across repetitions.

To compare needle types, we performed between group analyses separately for each predefined segment of the injection process. Because pressure distributions across needles did not satisfy normality, we used the Kruskal–Wallis test for each segment, followed by Dunn pairwise comparisons. Step up FDR adjustment recalibrated significance metrics, limiting expected proportion spurious positives among follow up contrasts.

## 3. Results

**Figure 3:**
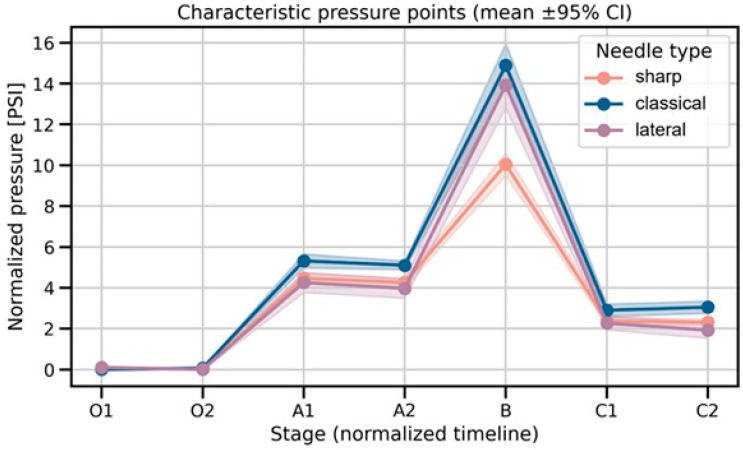
Line plots representing the averaged normalized pressure profiles (mean ± 95% CI) at identified characteristic points (O1, O2, A1, A2, B, C1, C2). The O1 to O2 section represents a pressure plateau attributed to resistance within the measurement system. Tissue injection was initiated at O2, where relative pressure was set to 0. The A1 to A2 section indicates intramuscular needle movement. Point B denotes contact of the needle tip with fascia. The C1 to C2 section indicates needle tip positioning within the interfascial space with hydrodissection. PSI denotes lbf/in^2^.

This part of the results section is presented in Figure 3 and Table 1.

- **Classical needle** (*Pajunk SonoPlex II*)

For the classical needle, all analyzed pressure distributions conformed to normality according to the Shapiro–Wilk test. Repeated measures ANOVA revealed highly significant differences between the mean pressures observed during the intramuscular injection phase, at the moment of fascial puncture, and during the interfascial phase (*p* = 4.73 × 10^−32^, effect size = 0.54). Post hoc comparisons indicated distinct patterns between all pairs of phases. The intramuscular and interfascial pressures differed strongly (adjusted *p* = 1.50 × 10^−15^, effect size = 3.50), as did the intramuscular and fascial puncture pressures (adjusted *p* = 2.34 × 10^−16^, effect size = −3.95). The greatest difference was observed between the interfascial and fascial puncture phases (adjusted *p* = 2.76 × 10^−17^, effect size = −4.98), indicating substantial shifts in pressure dynamics across the injection process.

Analysis of pressure variability demonstrated that the standard deviation of pressures differed significantly between the intramuscular and interfascial phases (*p* = 2.16 × 10^−3^, effect size = 0.73), reflecting greater instability in one phase relative to the other. The comparison of mean slopes between these two phases also showed a significant difference (*p* = 2.44 × 10^−5^, effect size = −1.31), suggesting distinct rates of pressure change during injection. Trend analysis further revealed a weak but significant decreasing tendency during the intramuscular phase (*p* = 4.63 × 10^−2^), while the interfascial phase exhibited a strong increasing trend (*p* = 1.56 × 10^−5^), indicating opposite pressure evolution patterns within the same injection sequence.

- **Lateral needle** (*Pajunk SonoPlex II Sprotte*)

For the lateral needle, all pressure distributions met the assumption of normality according to the Shapiro–Wilk test. Repeated measures ANOVA revealed highly significant differences among the mean pressures recorded during the intramuscular injection phase, at the point of fascial puncture, and during the interfascial phase (*p* = 1.54 × 10^−37^, effect size = 0.53). Post hoc pairwise comparisons confirmed that all phases differed significantly. The difference between intramuscular and interfascial pressures was substantial (*p* = 5.01 × 10^−19^, effect size = 1.93), while both phases also differed markedly from the fascial puncture phase (intramuscular vs fascial puncture: *p* = 5.01 × 10^−19^, effect size = −3.51; interfascial vs fascial puncture: *p* = 3.92 × 10^−20^, effect size = −4.42). These findings indicate pronounced shifts in mean pressure profiles between injection stages.

Analysis of pressure variability showed a highly significant difference in the standard deviation of pressures between the intramuscular and interfascial phases (*p* = 8.01 × 10^−6^, effect size = 1.20), indicating greater variability in one of these phases. Comparison of mean slopes revealed a moderate but significant difference between the same phases (*p* = 3.01 × 10^−2^, effect size = −0.53), reflecting changes in the dynamics of pressure buildup and release. Trend analysis demonstrated a strong decreasing tendency during the intramuscular phase (*p* = 5.0 × 10^−4^), whereas no significant trend was observed during the interfascial phase (*p* = 4.38 × 10^−1^), suggesting that pressure stabilization occurred following initial injection decline.

- **Sharp needle** (*Pajunk Sonoplex II Facet S*)

For the sharp needle, the Shapiro–Wilk test indicated that the pressure distributions deviated from normality. The Friedman test demonstrated highly significant differences among the mean pressures recorded during the intramuscular injection phase, at the point of fascial puncture, and during the interfascial phase (*p* = 3.44 × 10^−14^). Post hoc comparisons confirmed significant contrasts between all phases, with particularly large effect sizes. The difference between intramuscular and interfascial pressures was strong (*p* = 9.31 × 10^−10^, effect size = 4.48), as was the difference between the intramuscular and fascial puncture phases (*p* = 9.31 × 10^−10^, effect size = −5.08). The greatest contrast was observed between the interfascial and fascial puncture pressures (*p* = 9.31 × 10^−10^, effect size = −7.10), underscoring pronounced pressure transitions across the injection stages.

Analysis of pressure variability revealed a highly significant difference in the standard deviation of pressures between the intramuscular and interfascial phases (*p* = 1.77 × 10^−8^, effect size = 1.29), suggesting substantial differences in pressure stability. The comparison of mean slopes between these phases also indicated a significant difference (*p* = 4.95 × 10^−4^, effect size = −1.04), reflecting divergent rates of pressure change during injection. Trend analysis showed a clear decreasing pattern in the intramuscular phase (*p* = 5.0 × 10^−4^) and a strong increasing pattern in the interfascial phase (*p* = 4.0 × 10^−4^), indicating that the sharp needle produced distinctly opposite pressure evolutions in successive phases of the injection process.

- **Differences between needles**

The Kruskal–Wallis analyses revealed significant differences among the three needle geometries for all examined injection phases.

**Figure 4:**
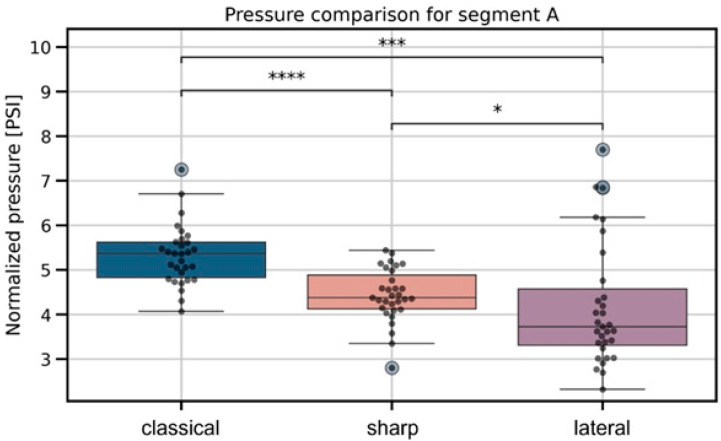
Boxplots illustrating intramuscular injection pressures for the three needle types (classical, lateral, and sharp). Significant differences identified by the Kruskal–Wallis test are marked with pairwise significance annotations following the convention: *p* ≤ 1 × 10^−4^ (****), *p* ≤ 1 × 10^−3^ (***), and *p* ≤ 5 × 10^−2^ (*).

For the intramuscular injection phase (Figure 4), the overall difference between needle types was highly significant (*p* = 2.0 × 10^−6^). Post hoc comparisons indicated that mean pressures for the lateral and classical needles differed substantially (*p* = 1.43 × 10^−4^, effect size = −1.04), while the classical needle also produced markedly higher pressures compared to the sharp needle (*p* = 9.0 × 10^−6^, effect size = 1.40). The difference between the lateral and sharp needles was smaller but still significant (*p* = 2.26 × 10^−2^, effect size = −0.24). These results indicate that the classical needle generated the highest intramuscular pressures, with the sharp and lateral needles producing progressively lower values.

**Figure 5:**
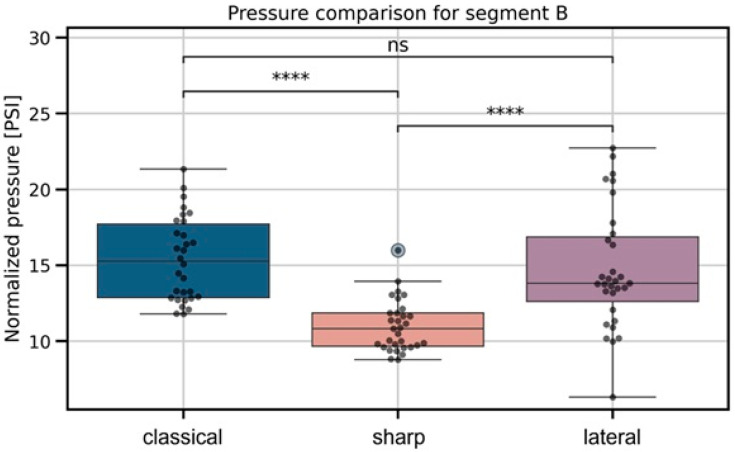
Boxplots showing pressure values at the moment of fascial puncture for the three needle geometries. Significant differences identified by the Kruskal–Wallis test are marked with pairwise significance annotations following the convention: *p* ≤ 1 × 10^−4^ (****), ns—not significant.

During the fascial puncture phase (Figure 5), the Kruskal–Wallis test again showed a strong overall effect (*p* = 7.52 × 10^−8^). Pairwise comparisons demonstrated that both the lateral and classical needles produced significantly higher pressures during fascia puncture than the sharp needle (lateral vs sharp: *p* = 6.66 × 10^−6^, effect size = 1.21; classical vs sharp: *p* = 4.71 × 10^−7^, effect size = 1.51), whereas no significant difference was observed between the lateral and classical needles (*p* = 6.70 × 10^−1^, effect size = −0.09).

**Figure 6:**
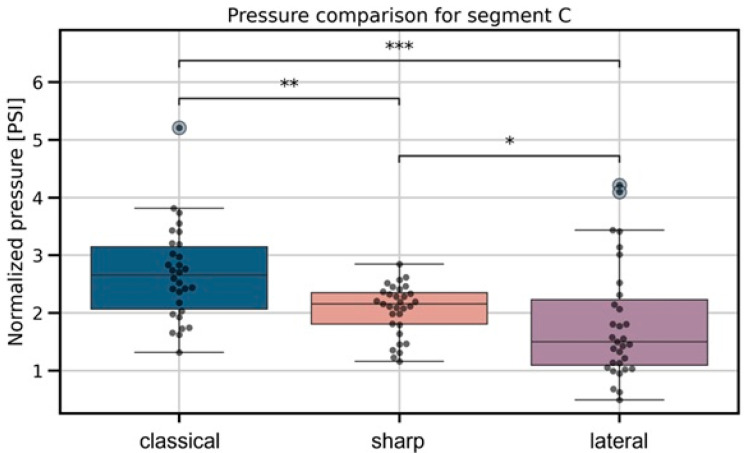
Boxplots representing interfascial injection pressures across needle types. Significant differences identified by the Kruskal–Wallis test are marked with pairwise significance annotations following the convention: *p* ≤ 1 × 10^−3^ (***), *p* ≤ 1 × 10^−2^ (**), and *p* ≤ 5 × 10^−2^ (*).

For the interfascial phase (Figure 6), significant differences were also found among needle types (*p* = 9.2 × 10^−5^). Post hoc analysis revealed that the lateral needle generated lower pressures compared to both the classical (*p* = 8.56 × 10^−4^, effect size = −0.93) and sharp needles (*p* = 2.26 × 10^−2^, effect size = −0.31), while the classical needle produced slightly higher interfascial pressures compared to the sharp needle (*p* = 1.80 × 10^−3^, effect size = 0.94).

## 4. Discussion

In the past, some regional compartmental blockades, such as TAPB, were performed using the decrease in resistance on the needle that is felt when passing through the fascial spaces. Even today, when ultrasound guidance is used it is not always possible for the operator to be sure if the needle tip is in the interfascial space [18]. To our knowledge, no studies have compared continuous injection pressures when using same diameter needles but with different types of tips. However, we have found articles that at least partially mention pressure data obtained during passage through tissues. In our study, we found some similarities to data of Roberto D. et al. [16], as their maximal injection pressure observed during indenting fascia was 119.55 kPa (=17.34 PSI) and pressures in troughs (as it was named in the article), which were in fact intramuscular pressures, were very similar to our data (30.99 kPa = 4.49 PSI) as is shown in Figure 4. Findings congruent with current results appear within publication by Steinfeldt T. et al. [19]; investigators used a needle tip pressure monitoring system analogous to that proposed by Quadri, conducting the study on cadavers. They yielded an average perineural injection pressure measured at the needle tip of 2.3 PSI (exactly in the range of our study, as shown in Figure 6, =15.86 kPa). Referring to fascia-indentation pressure, in the study of Capdevila M. et al. [14], authors stated that this pressure was measured with a pressure sensor that consisted of a Fabbri-Perrot optical cavity connected via an optic fiber to the control unit when using different injection flows (including 10 mL/min—identical to our study). Authors found that average indenting fascia pressure was 675 mmHg (=13.05 PSI, =89.98 kPa) which was similar to our findings (Figure 5). The mentioned studies used pressure optic fiber sensor mounted inside needle bore just after the tip, not continuous injection pressure monitoring, thus they are comparable in results but have completely different methodology.

Our study has several limitations: it was conducted on animal material (not human tissue) and was performed post-mortem on pork thigh. Porcine thigh differs substantially from living human tissue in terms of perfusion, elasticity, and fascial compliance; as a result, our study cannot be extrapolated to humans nor to clinical settings, mainly because of the lower compliance of porcine tissues—especially fascia [20,21].

At the same time, our study has several strengths: we used needles from the same company, with the same diameter, while continuously measuring pressure. The latter compensated for the limitations of the [14] study, while increasing the range of measurements to 30 per needle type.

The results obtained during our study clearly indicate that needles of the same diameter but with different tips generate different injection pressures when passing through tissues. This finding indicates that further research is needed on needles that differ not only in diameter but also in tip, including cadaver and clinical studies.

It seems that when developing a system to support physicians in performing regional blocks, the technical properties of individual needles for regional blocks should be considered on a case-by-case (individual) basis. Only in this way will it be possible to create an efficient and reliable measurement system which, in combination with ultrasound, might significantly facilitate and improve the safety of regional anesthesia procedures and pain management. Taking into account the correspondence by Adams A.M. [22], in our research we sought to identify a potential solution to the problem of pressure measurement during continuous administration of local anesthetic solutions in such devices, and to emphasize that the problem is complex and requires separate studies for needles of different sizes and with different tips.

Taking into consideration recent work by Istenič S. et al. [23], who quantified bupivacaine distribution within skeletal muscle and across fascial barriers using HPLC-MS in comparison to water-soluble dyes, like methylene blue, it may be stated, that there is clear discrepancy between the spread of bupivacaine and hydrophilic tracers such as methylene blue or iodinated contrast. As a result, in combination with our studies, this clearly shows that further, complex mechano-pharmacological studies are required in this field.

## 5. Conclusions

Together, these findings demonstrate that needle geometry substantially influences pressure dynamics throughout the injection process, with the classical design associated with the highest fascia-penetration injection pressures and the sharp needle exhibiting the lowest, while the lateral design is associated with the lowest intramuscular-penetration and interfascial pressures.

## Figures and Tables

**Table 1:** Consolidated account of inferential comparisons run per cannula type plus cross type contrasts, including significance markers aligned with customary notation: *p* ≤ 1 × 10^−4^ (****), *p* ≤ 1 × 10^−3^ (***), *p* ≤ 1 × 10^−2^ (**), and *p* ≤ 5 × 10^−2^ (*), ns—not significant.

	Needle		
	Classical	Lateral	Sharp
**Mean pressure (A–B–C)**	****	****	****
**Variability (A–C)**	**	****	****
**Slope (A–C)**	***	*	***
**Trend (A)**	↘	↘	↘
**Trend (C)**	↗	-	↗
**Intramuscular (A)**	****		
**Fascial puncture (B)**	****		
**Interfascial (C)**	****		

## Data Availability

Data are attached as Supplementary Materials in a separate file.

## Supplementary Materials

The following supporting information can be downloaded at: https://www.mdpi.com/article/10.3390/jcm15041458/s1. Additional data files for the classic, lateral, and sharp needles.
